# 

**Published:** 1908-05-09

**Authors:** 


### BOOKS RECEIVED.

T. Fisher Unwin.

"Health at its Best *v.* Cancer and Other Diseases." By Robt. Bell, M.B.

Cassell and Co.

"Diseases of the Nervous System." By H. Campbell Thomson, M.D.

"Electrical Treatment." By Wilfred Harris, M.D.

J. and A. Churchill.

"Hernia: Its Cause and Treatment." By R. W. Murray, F.R.C.S.

### FORTHCOMING MEDICAL BOOKS.

Messrs. Cassell and Co. are about to issue a new and revised edition of "Hygiene and Public Health," by Dr. B. A. Whitelegge and Dr. G. Newman, and a new volume by Dr. G. E. Herman, entitled "The Student's Handbook of Gynæcology."

Messrs. Henry Frowde and Hodder and Stoughton will publish during May the second edition of "Operations of General Practice," by Mr. Edred M. Corner and Dr. H. I. Pinches; also the following new books: "A System of Syphilis," Vol. I., edited by Mr. D'Arcy Power and Mr. J. Keogh Murphy; "The Writings of the late Sir William Broadbent, F.R.S.," collected and edited by Dr. Walter Broadbent; "Glandular Enlargements and other Diseases of the Lymphatic System," by Mr. Arthur Edmunds; "The Law in General Medical Practice, or Clinical Jurisprudence," by Dr. Stanley B. Atkinson; "Diseases of the Eye," by Mr. Stephen Mayou; and "Gunshot Wounds," by Major C. G. Spencer, R.A.M.C.

The above publishers also have in active preparation "Heart Diseases," by Dr. James Mackenzie, and "The Rectum; Its Diseases and Developmental Defects," by Sir Charles Ball, F.R.S.

### THE GRADUATES' MEDICAL SCHOOLS.

DIARY FOR WEEK MAY 9 to 16.

**NORTH-EAST LONDON POST-GRADUATE COLLEGE, Prince of Wales's General Hospital, Tottenham, N.**

**At 4.30 p.m.**
**May 14,** Mr. Walter Edmunds, **Surgical Demonstration.**

**THE THROAT HOSPITAL, Golden Square, W.**

The Summer Course of Lectures will deal with **Inflammatory Affections of the Upper Air Passages.** The lectures will be at **5.30 p.m.,** on Mondays and Thursdays, beginning on **May 18.**

**CHARING CROSS HOSPITAL, W.C.**

Post-Graduate Demonstrations.

**At 4 p.m.**
**May 14,** Dr. Galloway, **Medical.**

**THE POST-GRADUATE COLLEGE, West London Hospital, Hammersmith, W.**

**At 12 noon.**
**May 11,** Dr. Low, **Pathological Demonstration.**
**At 12.15 p.m.**
**May 13,** Dr. Pritchard, **Practical Medicine.**
**At 5 p.m.**
**May 11,** Dr. Davis, **Diseases of the Pharynx.**
**May 12,** Mr. Pardoe, **Stricture of the Urethra.**
**May 13,** Dr. Beddard, **Medicine, I.**
**May 14,** Dr. Davis, **Eustachian Obstruction and its results.**
**May 15,** Dr. Pritchard, **Clinical Pathology as an aid to Diagnosis.**

**MEDICAL GRADUATES' COLLEGE AND POLYCLINIC, Chenies Street, W.C.**

**At 5.15 p.m.**
**May 11,** Dr. J. E. Squire, **Hæmoptysis: its Significance and Treatment.**
**May 12,** Dr. G. F. Still, **Infantile Marasmus.**
**May 13,** Mr. W. H. Clayton-Greene, **Abdominal Injuries.**
**May 14,** Dr. C. O. Hawthorne, **The Diagnosis of Functional Nervous Disease.**

**THE HOSPITAL FOR SICK CHILDREN, Great Ormond Street, W.C.**

**At 4 p.m.**
**May 14,** Mr. H. S. Collier, **Some Minor Operations in Children.**

**NATIONAL HOSPITAL FOR THE PARALYSED AND EPILEPTIC, Queen Square, Bloomsbury, W.C.**

**At 3.30 p.m.**
**May 12,** Dr. G. Stewart, **Syphilis of the Nervous System.**
**May 15,** Dr. G. Stewart, **Syphilis of the Nervous System.**

